# MLN4924 Exerts a Neuroprotective Effect against Oxidative Stress via Sirt1 in Spinal Cord Ischemia-Reperfusion Injury

**DOI:** 10.1155/2019/7283639

**Published:** 2019-04-17

**Authors:** Sifei Yu, Lei Xie, Zhuochao Liu, Changwei Li, Yu Liang

**Affiliations:** ^1^Department of Orthopedics, Ruijin Hospital, Shanghai Jiao Tong University School of Medicine, 197 Ruijin 2nd Road, Shanghai 200025, China; ^2^Shanghai Key Laboratory for Prevention and Treatment of Bone and Joint Diseases with Integrated Chinese-Western Medicine, Shanghai Institute of Traumatology and Orthopedics, Ruijin Hospital, Shanghai Jiao Tong University School of Medicine, 197 Ruijin 2nd Road, Shanghai 200025, China

## Abstract

Oxidative stress is a leading contributor to spinal cord ischemia-reperfusion (SCIR) injury. Recently, MLN4924, a potent and selective inhibitor of the NEDD8-activating enzyme, was shown to exert a neuroprotective effect against oxidative stress *in vitro*. However, it is unknown whether MLN4924 plays a protective role against SCIR injury. In the present study, we found that MLN4924 treatment significantly attenuated oxidative stress and neuronal cell death induced by H_2_O_2_ in SH-SY-5Y neural cells and during rat SCIR injury. Furthermore, MLN4924 administration restored neurological and motor functions in rats with SCIR injury. Mechanistically, we found that MLN4924 protects against H_2_O_2_- and SCIR injury-induced neurodegeneration by regulating sirtuin 1 (Sirt1) expression. Collectively, these findings demonstrate the neuroprotective role of MLN4924 against oxidative stress in SCIR injury via Sirt1.

## 1. Introduction

Spinal cord ischemia-reperfusion (SCIR) injury is a major complication of thoracoabdominal aortic surgery [1], which can result in debilitating paraplegia with a reported incidence of 3-18% [2]. Despite considerable therapeutic interventions to reduce SCIR injury, the protective effect of these interventions is very limited and the incidence of paraplegia remains high [3, 4].

The pathophysiological changes underlying ischemia-reperfusion (I/R) injury involve necrosis and apoptosis [5]. During I/R, hemorrhage and fluid resuscitation promote the excessive production of reactive oxidative species (ROS), which overwhelms the antioxidant defense system and ultimately leads to cell death and neuronal damage [6]. Although the cellular and molecular mechanisms that cause I/R damage to the medulla spinalis are poorly understood, many studies have demonstrated that oxidative stress plays a crucial role in the pathogenesis of I/R injury [7, 8]. In line with these reports, our previous studies revealed that the administration of adenine dinucleotide (NAD) or hydrogen sulfide effectively protected against SCIR injury by reducing oxidative stress-induced neuronal apoptosis [1, 9, 10]. Thus, pharmacological therapies targeting oxidative stress may be critical for limiting SCIR injury.

MLN4924, a newly discovered small molecule inhibitor of the NEDD8-activating enzyme (NAE), inactivates Cullin-RING E3 ubiquitin ligases (CRLs) by blocking CULLIN neddylation [11]. In addition to the antiproliferative and proapoptotic properties [12, 13], the neuroprotective role of MLN4924 was also demonstrated. It was reported that MLN4924 significantly attenuated H_2_O_2_-induced neurocyte damage *in vitro* via Nrf2 protein accumulation [14]. However, it is unknown whether MLN4924 effectively prevents oxidative damage in SCIR.

Given that oxidative stress contributes to neurodegeneration during SCIR and MLN4924 is reported to provide neuroprotection against oxidative injury, we aimed to investigative whether MLN4924 could protect against oxidative stress-induced cell damage during SCIR.

## 2. Materials and Methods

### 2.1. Animals

Eight-week-old male Sprague-Dawley rats, weighing 180-250 g, were obtained from Shanghai Laboratorial Animal Center at the Chinese Academy of Sciences. The rats had ad libitum access to food and water in an air-conditioned room with a 12 h light-dark cycle, at 25°C and 50% relative humidity, in the animal cage at Ruijin Hospital, Shanghai Jiao Tong University School of Medicine, China.

### 2.2. Ethics Statement

All animal experiments were performed in accordance with the protocol approved by the Shanghai Jiao Tong University (SJTU) Animal Care and Use Committee (IACUC protocol number: SYXK (Shanghai) 2011-0113) and in accordance with the Ministry of Science and Technology of the People's Republic of China Animal Care guidelines. All surgeries were performed under anesthesia, and all efforts were made to minimize the suffering of animals.

### 2.3. SCIR Injury

The I/R model was generated according to previous reports [1, 9, 10]. All rats were neurologically intact and anesthetized with intraperitoneal injection of 2.5% sodium pentobarbital (60 mg/kg) following SCIR injury. After the procedures and drug interventions, the animals were euthanized with an overdose of sodium pentobarbital (intraperitoneal injection, 160 mg/kg) [15, 16].

### 2.4. SCIR Treatment

For the *in vivo* experiments, the animals were assigned a unique number and then randomly divided into groups (*n* = 6 per group). The sham group underwent the surgical procedure without aortic clipping. The I/R group received abdominal aortic exposure and cross-clamping for 60 min followed by intraperitoneal injection of an equivalent volume of 0.9% saline solution immediately after reperfusion. The rats in the I/R + MLN4924 (S7109, Selleck Chemicals) (*n* = 6) and I/R + MLN4924 + EX527 (S1541, Selleck Chemicals) (*n* = 6) groups also received the same surgical procedure as those in the I/R group but were treated with MLN4924 immediately after I/R injury. EX527 was injected 0.5 h before the onset of SCIR in the I/R + MLN4924 + EX527 group. All experiments were repeated three times.

### 2.5. Neurological Function Assessment

Locomotor recovery after SCIR was assessed using the Basso, Beattie, and Bresnahan (BBB) open-field locomotor scale [17], which is scored from 0 (complete paralysis) to 21 (normal locomotion). The BBB scores were recorded at 1, 6, 12, and 24 h in the acute phase after reperfusion by two experienced investigators who were blinded to the experimental groups and treatments. Disagreements were resolved via discussion to reach a consensus.

### 2.6. Cell Culture

SH-SY-5Y neural cells [18, 19] were purchased from the China Center for Type Culture Collection (Wuhan University, China, 22-4-2015, http://www.cctcc.org) and cultured in Dulbecco's modified Eagle's medium (DMEM) (Invitrogen, Carlsbad, CA, USA) containing 10% fetal bovine serum (Invitrogen) and antibiotics (100 units/mL penicillin and 100 *μ*g/mL streptomycin) incubated in a humidified atmosphere containing 5% CO_2_ at 37°C. The cells were passaged every 3-5 days with 0.25% trypsin-ethylenediaminetetraacetic acid (Invitrogen, Carlsbad, CA, USA).

### 2.7. Cell Treatment

A variety of freshly prepared doses of H_2_O_2_ were added for 6 h to the cells with or without pretreatment with MLN4924 for 1 h before all experiments. The neurotoxic effects of H_2_O_2_ and MLN4924 were assessed by cell counting kit-8 analysis, terminal deoxynucleotidyl transferase-mediated dUTP nick end labeling (TUNEL) immunofluorescence staining, and immunoblot analysis of Bax, B-cell lymphoma 2 (BCL2), and cleaved caspase-3 expression. The oxidative stress was detected by malondialdehyde (MDA) concentration and superoxide dismutase (SOD) activity.

### 2.8. Oxidative Stress Assay

SOD activity and the MDA concentration in the SH-SY-5Y cells and spinal cord tissue were determined using a superoxide dismutase assay kit (Beyotime, Shanghai, China) and MDA kit (Nanjing Jiancheng Bioengineering Institute, China). The SH-SY-5Y cells were pretreated with different concentrations of MLN4924 for 1 h prior to exposure to H_2_O_2_ (200 *μ*M) for an additional 6 h. The cells were washed twice with precooled phosphate-buffered saline (PBS). Fresh spinal cord tissues were collected and washed with precooled PBS. We added ninefold the mass of PBS to convert the spinal cord tissue into 100 g/L homogenates in a homogenizer. The homogenates were centrifuged at 4°C for 15 min at a speed of 3500 r/min. The homogenates were collected and incubated in radioimmunoprecipitation assay (RIPA) lysis buffer in order to determine the total protein contents using the bicinchoninic acid protein assay kit (Beyotime Biotechnology). Samples were measured and analyzed according to the manufacturer's instructions.

### 2.9. Immunofluorescence Staining of Sirtuin 1

The treated SH-SY-5Y cells were fixed with 4% paraformaldehyde for 15 min at 22 ± 2°C, then permeabilized with 0.5% Triton X-100 for 10 min, and finally blocked with 5% serum albumin for 1 h. The cells were then incubated with primary antisirtuin 1 (Sirt1) (1 : 100; Abcam) diluted in 1% bovine serum albumin at 4°C overnight. The cells were washed three times with PBS with Tween 20 (PBST) and incubated with the secondary antibody solution for 1 h at 22 ± 2°C in the dark and then washed three times with PBST. DAPI was added dropwise, and the cells were incubated in the dark for 5 min to stain the nuclei. The excess DAPI was removed by washing three times with PBST, the antifluorescence quencher was added dropwise, and images were collected under a fluorescence microscope.

### 2.10. Immunohistochemical Staining Analysis of NEDD8 and CULLIN1

Immunohistochemical staining was performed as previously described [20]. Briefly, after the slides were incubated with blocking serum for 60 min, they were blotted and then overlaid with the primary antibody against NEDD8 (NEDD8 (19E3) rabbit monoclonal antibody #2754, CST), CULLIN1 (rabbit monoclonal antibody (EPR3103Y) to CULLIN1/CUL-1, ab75817, Abcam), or Sirt1 (rabbit monoclonal antibody (EPR18239) to Sirt1, ab189494, Abcam) for 2 h at 22 ± 2°C. Subsequently, biotinylated secondary antibodies (anti-rabbit IgG (H + L), biotinylated antibody #14708, CST) were added into the sections, followed by a peroxidase-labeled streptavidin-biotin staining technique (DAB Kit, Invitrogen, Paisley, UK).

### 2.11. siRNA Preparation and Targeting Gene Knockdown

siRNA oligonucleotides encoding human *Sirt1* and the scrambles were designed and synthesized by GenePharma (Shanghai, China). A blast search was performed using the National Center for Biotechnology Information (NCBI) database to ensure that the siRNA constructs targeted only human *Sirt1*. A mixture of three siRNA oligonucleotides for *Sirt1* was used to transfect the SH-SY-5Y cells. The siRNA construct and transfection were performed according to the manufacturer's protocol of Lipofectamine 3000 (Invitrogen). After 24 h of transfection, the cells were treated with H_2_O_2_/MLN4924.

### 2.12. TUNEL Staining

A TUNEL assay was performed to detect cell apoptosis. The cells were stained using the In Situ Cell Death Detection kit (Roche Diagnostics GmbH) according to the manufacturer's instructions. Briefly, the cells were incubated with TUNEL solution containing TMR-dUTP for 1 h at 37°C. After labeling with TUNEL, the cell nuclei were counterstained with DAPI. The cells were then observed under a fluorescence microscope, and images were acquired.

### 2.13. Immunoblotting Analysis

The spinal cords and SH-SY-5Y cells as described were lysed using RIPA buffer (pH 7.4) containing protease inhibitor cocktail (Roche). For the Western blot, 10 *μ*g of total protein was used. Sirt1, acetylated forkhead box O1 (FoxO1), Bax, BCL2, and cleaved caspase-3 were detected by immunoblotting with Sirt1 antibodies (Abcam), acetylated FoxO1 (Abcam), Bax (Cell Signaling Technology), BCL2 (Cell Signaling Technology), and cleaved caspase-3 (Cell Signaling Technology), respectively. The intensities of the protein bands were quantified by densitometry analysis using NIH ImageJ software.

### 2.14. Annexin V-FITC/PI Analysis by Flow Cytometry

The cells were stained with Annexin V and PI Kit (BD Pharmingen™ FITC Annexin V Apoptosis Detection Kit). The analysis was performed according to the manufacturer's instructions. Briefly, the treated SH-SY-5Y cells were washed with PBS twice and resuspended in 1x binding buffer at a concentration of 1 × 10^6^ cells/mL. Then, 5 *μ*L of Annexin V and 5 *μ*L of PI were added to the 1x binding buffer and the solution was incubated for 15 min at 22 ± 2°C in the dark. The cells were analyzed using CytoFLEX (Beckman Coulter Inc., Brea, CA, USA) and CytExpert (version 2.1; Beckman Coulter Inc.).

### 2.15. Statistical Analysis

All data are presented as the mean ± standard error of the mean. Two-tailed *t*-tests were used to determine the significances between two groups. We performed analyses of multiple groups using a one-way ANOVA with a Bonferroni posttest in GraphPad Prism 5. For all statistical tests, *P* values of <0.05 were considered statistically significant.

## 3. Results

### 3.1. Neddylation Might Be Activated in SCIR Injury

**Figure 1:**
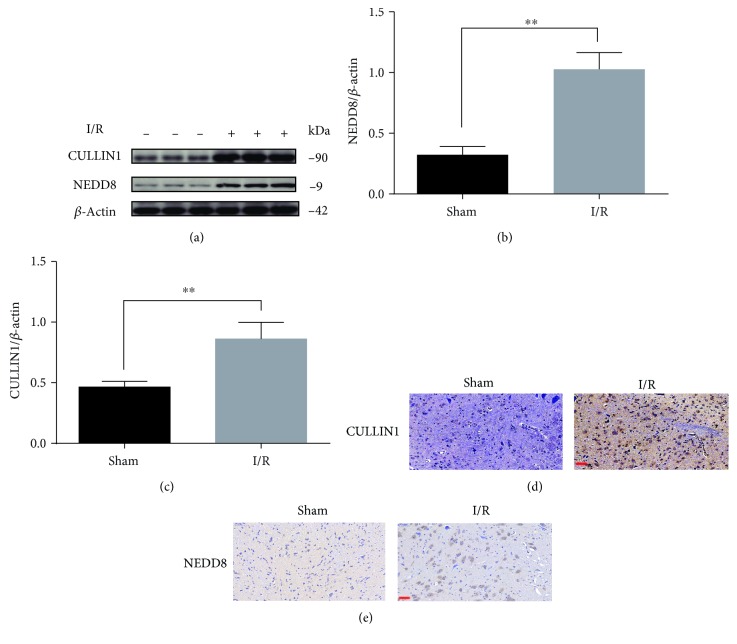
NEDD8 and Cullin1 were notably increased in the spinal cord after IR injury. (a–c) Western blot analysis of NEDD8 and Cullin1 expression in the spinal cord of rats after SCIR injury. (d, e) Immunohistochemical staining analysis of Cullin1 and NEDD8 expression in the spinal cord of rats after SCIR injury. ^∗∗^ *P* < 0.01. *P* values were analyzed by two-tailed *t*-tests. Scale bar represents 50 *μ*m.

MLN4924 is reported to inactivate CRLs by blocking CULLIN neddylation. Therefore, to determine whether MLN4924 can effectively prevent oxidative damage in SCIR injury, the presence of neddylation activation-related genes, such as *NEDD8* and *CULLIN1* [20], was evaluated in the spinal cords of rats with or without I/R injury. The results of Western blot (Figures 1(a)–1(c)) and immunohistochemical staining (Figures 1(d) and 1(e)) analyses revealed that CULLIN1 and NEDD8 were notably increased in the spinal cord after I/R injury. This finding indicates that neddylation activation might play a pathogenic role in SCIR injury and provides a strong rationale for evaluating the therapeutic effect of MLN4924.

### 3.2. MLN4924 Ameliorated H_2_O_2_-Induced Oxidative Stress and Apoptosis in SY-SH-5Y Neural Cells

**Figure 2:**
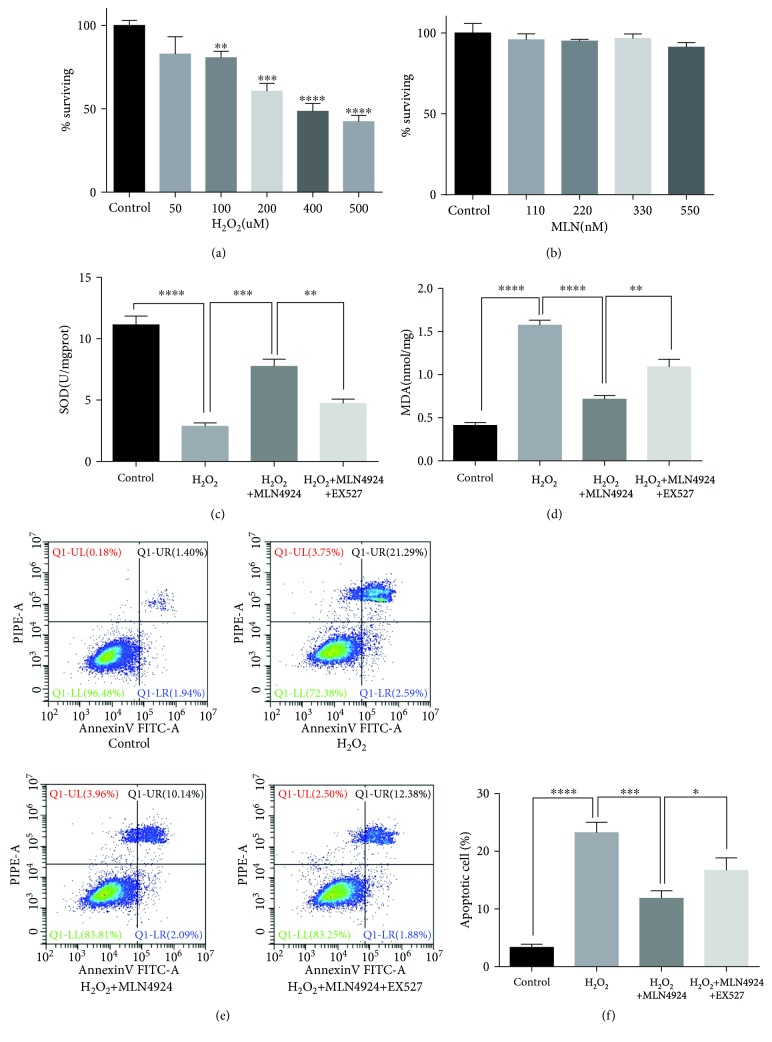
MLN4924 protects against H_2_O_2_-induced oxidative stress and cell death in SH-SY5Y cells via Sirt1. Cell proliferation in response to different doses of either (a) H_2_O_2_ or (b) MLN4924 was detected with a cell counting kit-8 (CCK8) assay. (c) SOD activity and (d) MDA concentration in SH-SY5Y cells treated with or without H_2_O_2_ and MLN4924/EX527. (e, f) FACS analysis of apoptosis in SH-SY5Y cells treated with or without H_2_O_2_ and MLN4924/EX527. H_2_O_2_: 200 *μ*M; MLN4924: 110 nM; Ex527: 1 *μ*M. ^∗^ *P* < 0.05, ^∗∗^ *P* < 0.01, ^∗∗∗^ *P* < 0.001, and ^∗∗∗∗^ *P* < 0.0001. *P* values were analyzed by one-way analysis of variance (ANOVA).

**Figure 3:**
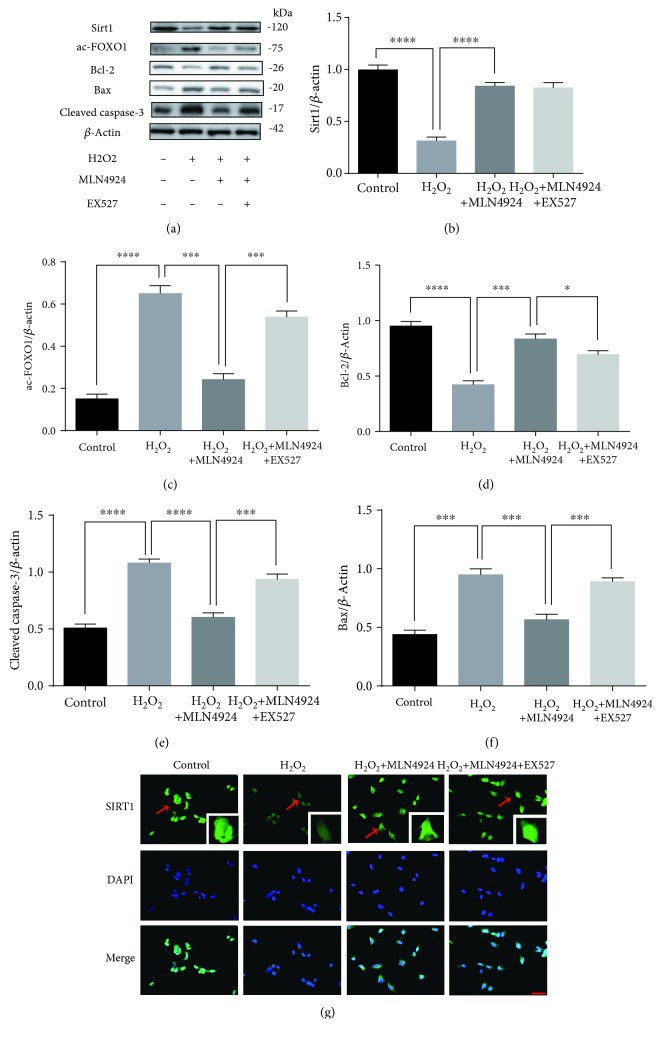
MLN4924 protects against H_2_O_2_-induced apoptosis of SH-SY5Y cells via Sirt1. (a–f) Western blot analysis of Sirt1, acetylated FoxO1, BCL2, Bax, and cleaved caspase-3 in SH-SY5Y cells treated with or without H_2_O_2_ and MLN4924/EX527. (g) Immunostaining of Sirt1 in SH-SY5Y cells treated with or without H_2_O_2_ and MLN4924/EX527. H_2_O_2_: 200 *μ*M; MLN4924: 110 nM; Ex527: 1 *μ*M. ^∗^ *P* < 0.05, ^∗∗∗^ *P* < 0.001, and ^∗∗∗∗^ *P* < 0.0001. *P* values were analyzed by one-way ANOVA. Scale bar represents 50 *μ*m.

The neuroprotective role of MLN4924 against H_2_O_2_-induced oxidative damage was first demonstrated in SY-SH-5Y neural cells. Figures 2(a) and 2(b) present the results of the cell counting kit-8 assay. H_2_O_2_ treatment inhibited cell viability in a dose-dependent manner (Figure 2(a)), whereas exposure to different concentrations of MLN4924 had no significant effect on the viability of SY-SH-5Y neural cells (Figure 1(b)). Therefore, 200 *μ*M H_2_O_2_ and 110 nM MLN4924 were used in our subsequent *in vitro* experiments. The concentration of MDA and enzymatic activities of SOD are often used to evaluate oxidative and antioxidative reactions [21]. A decrease in SOD activity (Figure 2(c)) and an increase in MDA concentration (Figure 2(d)) were detected in SY-SH-5Y neural cells in response to H_2_O_2_ treatment. However, both processes were significantly reduced by incubation with MLN4924 (Figures 2(c) and 2(d)). This indicates that MLN4924 successfully reduced H_2_O_2_-induced oxidative stress in SY-SH-5Y neural cells. In addition, the presence of cell apoptosis, identified by fluorescence-activated cell sorting (FACS) analysis (Figures 2(e) and 2(f)), and the expression of BCL2, Bax, and cleaved caspase-3 detected by Western blot (Figures 3(a)–3(f)) demonstrated that incubation with MLN4924 significantly decreased H_2_O_2_-induced death of SY-SH-5Y neural cells. Taken together, these data demonstrated that MLN4924 ameliorated H_2_O_2_-induced oxidative stress and apoptosis in SY-SH-5Y neural cells.

### 3.3. MLN4924 Ameliorated H_2_O_2_-Induced Oxidative Stress and Apoptosis in SY-SH-5Y Neural Cells via Sirt1

**Figure 4:**
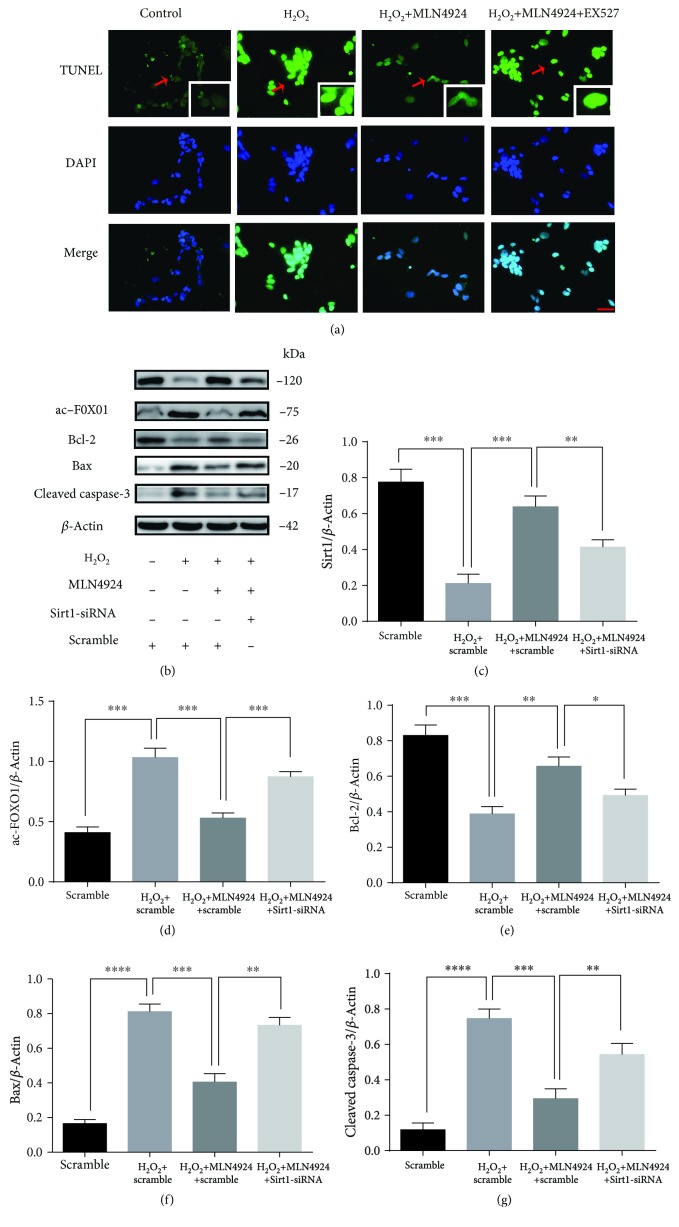
MLN4924 protects against H_2_O_2_-induced apoptosis of SH-SY5Y cells via Sirt1. (a) TUNEL immunostaining in SH-SY5Y cells treated with or without H_2_O_2_ and MLN4924/EX527. (b–g) Western blot analysis of Sirt1, acetylated FoxO1, BCL2, Bax, and cleaved caspase-3 in SH-SY5Y cells treated with or without H_2_O_2_ and MLN4924/Sirt1-siRNA. H_2_O_2_: 200 *μ*M; MLN4924: 110 nM; Ex527: 1 *μ*M. ^∗^ *P* < 0.05, ^∗∗^ *P* < 0.01, ^∗∗∗^ *P* < 0.001, and ^∗∗∗∗^ *P* < 0.0001. *P* values were analyzed by one-way ANOVA. Scale bar represents 50 *μ*m.

In our previous studies, we demonstrated the protective effect of NAD against SCIR injury via reductions in oxidative stress-induced neuronal apoptosis [9, 10]. Sirt1 is a NAD-dependent protein deacetylase and has been demonstrated to protect against I/R injury in various organs [22–24]. Furthermore, our present results indicated that incubation with MLN4924 significantly restored Sirt1 expression, which was decreased by H_2_O_2_ (Figures 3(a) and 3(g)). Therefore, we hypothesized that the amelioration of H_2_O_2_-induced oxidative stress and apoptosis in SY-SH-5Y neural cells by MLN4924 might occur via Sirt1. To test this hypothesis, the selective Sirt1 inhibitor, EX527, and Sirt1 siRNA were used to inhibit the Sirt1 pathway. As illustrated in Figures 3(a) and 4(b), Sirt1 inhibition via 1 *μ*M EX527 or siRNA significantly increased acetylated FoxO1 accumulation. Accompanied by the increased oxidative stress, as evidenced by the decreased SOD activity (Figure 2(c)) and increased MDA concentration (Figure 2(d)), the protective effect of MLN4924 on H_2_O_2_-induced apoptosis of SY-SH-5Y neural cells was significantly blocked by incubation with EX527 or Sirt1 siRNA, as evidenced by the expression of BCL2, Bax, and cleaved caspase-3 as detected by Western blot (Figures 3(a)–3(f) and 4(b)–4(g)); the presence of TUNEL-positive cells (Figure 4(a)); and the FACS analysis of cellular apoptosis (Figures 2(e) and 2(f)). Taken together, these results demonstrated that MLN4924 ameliorated H_2_O_2_-induced oxidative stress and apoptosis in SY-SH-5Y neural cells via Sirt1.

### 3.4. MLN4924 Protects against Neurodestruction in SCIR Injury

**Figure 5:**
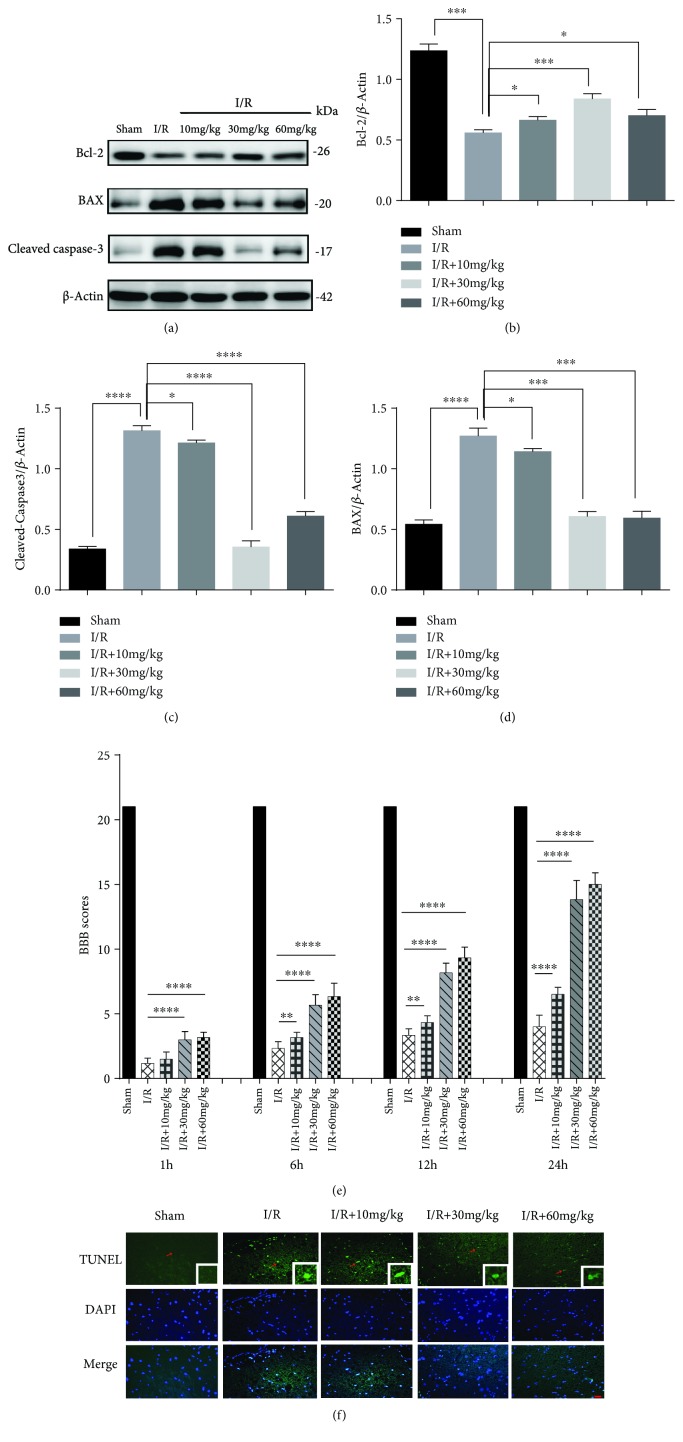
MLN4924 protects against neurodestruction in SCIR injury. (a–d) Western blot analysis of BCL2, Bax-2, and cleaved caspase-3 in the spinal cord of rats intraperitoneally injected with different doses of MLN4924. (e) The BBB scores of animals treated with different doses of MLN4924 after SCIR injury. *n* = 6. (f) TUNEL immunostaining in the spinal cord of rats which were intraperitoneally injected with different doses of MLN4924. ^∗^ *P* < 0.05, ^∗∗^ *P* < 0.01, ^∗∗∗^ *P* < 0.001, and ^∗∗∗∗^ *P* < 0.0001. *P* values were analyzed by one-way ANOVA. Scale bar represents 50 *μ*m.

We also investigated the *in vivo* neuroprotective role of MLN4924 in SCIR injury. Pretreatment with 10, 30, and 60 mg/kg of MLN4924 significantly decreased the apoptosis of neuronal cells as detected by BCL-2, Bax, and reduced caspase-3 expression and the number of TUNEL-positive cells (Figures 5(a)–5(d) and 5(f)). Furthermore, MLN4924 pretreatment restored neurological and motor functions as indicated by the BBB scores (Figure 5(e)). The results also indicated that 30 mg/kg of MLN4924 exerted a more protective effect than did 10 and 60 mg/kg of MLN4924. Taken together, these results demonstrated that 30 mg/kg of MLN4924 effectively protected against neurodestruction during SCIR injury, and thus, 30 mg/kg of MLN4924 was used in the subsequent experiments.

### 3.5. MLN4924 Protects against Neurodestruction in SCIR Injury via Sirt1

**Figure 6:**
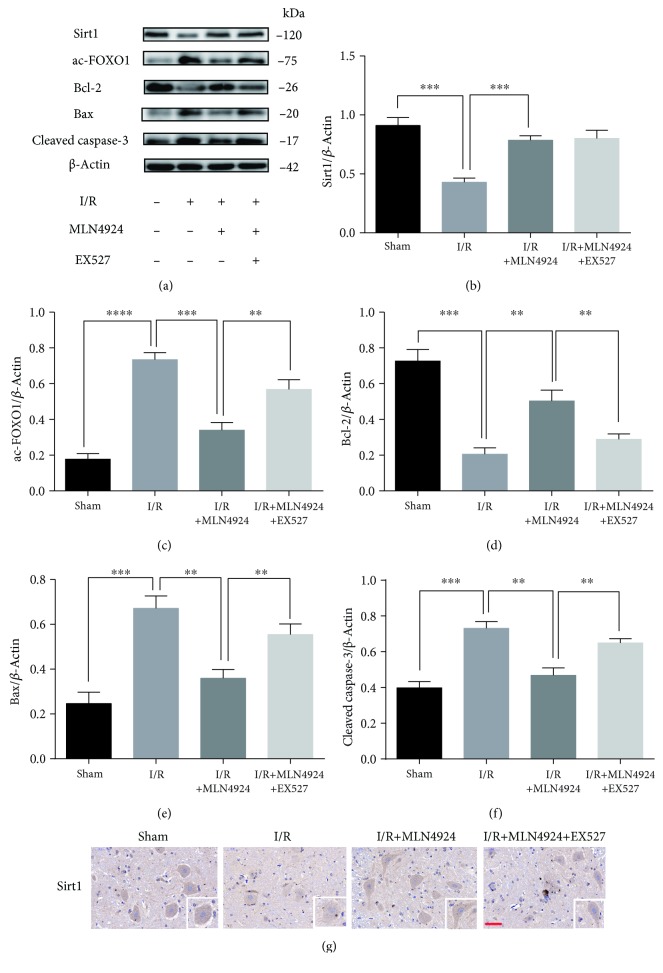
MLN4924 protects against neurodestruction in SCIR via Sirt1. (a–f) Western blot analysis of Sirt1, acetylated FoxO1, BCL2, Bax, and cleaved caspase-3 in the spinal cord of rats treated with or without MLN4924/EX527 after SCIR. (g) Immunohistochemical staining for Sirt1 in the spinal cord of rats treated with or without MLN4924/EX527 after SCIR. MLN4924: 30 mg/kg; Ex527: 1 mg/kg. ^∗∗^ *P* < 0.01, ^∗∗∗^ *P* < 0.001, and ^∗∗∗∗^ *P* < 0.0001. *P* values were analyzed by one-way ANOVA. Scale bar represents 50 *μ*m.

**Figure 7:**
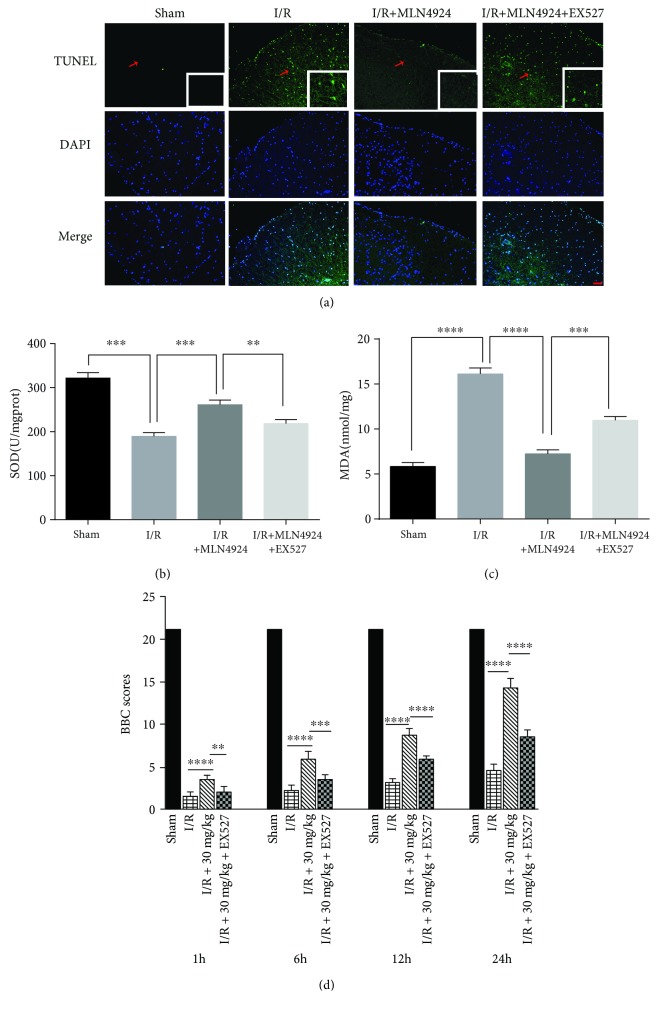
MLN4924 protects against neurodestruction in SCIR injury via Sirt1. (a) TUNEL immunostaining in the spinal cord of rats treated with or without MLN4924/EX527 after SCIR injury. (b) SOD activity and (c) MDA concentration in the spinal cord of rats treated with or without MLN4924/EX527 after SCIR. (d) The BBB scores of animals treated with or without MLN4924/EX527 after SCIR. *n* = 6. MLN4924: 30 mg/kg; Ex527: 1 mg/kg. ^∗∗^ *P* < 0.01, ^∗∗∗^ *P* < 0.001, and ^∗∗∗∗^ *P* < 0.0001. *P* values were analyzed by one-way ANOVA. Scale bar represents 50 *μ*m.

Having established the neuroprotective role of MLN4924 during SCIR, we next sought to investigate whether the protective role of MLN4924 was dependent on Sirt1 *in vivo*. The Western blot and immunohistochemical staining analyses revealed that Sirt1 expression was significantly decreased in neuronal cells after SCIR injury. However, MLN4924 treatment significantly restored Sirt1 expression (Figures 6(a), 6(b), and 6(j)). Consistent with the *in vitro* results, Sirt1 inhibition by EX527 significantly blocked the antiapoptotic (Figures 4(a)–4(f) and 7(a)) and antioxidative (Figures 7(b) and 7(c)) effects of MLN4924 on neuronal cells in SCIR injury. In addition, the ability of MLN4924 to rescue neurological and motor function in SCIR was also reduced by EX527 (Figure 7(d)). Taken together, these results demonstrated that MLN4924 protects against neurodestruction in SCIR via Sirt1.

## 4. Discussion

Although the pathophysiological mechanisms that underlie hypoxic/ischemic injury in the spinal cord have not been fully elucidated, the rapid increase in free radicals and oxidative stress is currently considered the most critical event for irreversible cellular damage in SCIR injury [25]. A reduction in blood flow in the spinal cord (ischemia) after aortic or spinal surgery causes hypoxia in the spinal cord and increases the levels of lactic acid, hypoxanthine, and lipid peroxide. Reperfusion restores lost cellular functions during ischemia; however, it increases blood flow and tissue oxygenation and thereby causes further damage in ischemic tissues via the formation of ROS. This leads to reperfusion injury [26, 27]. Therefore, therapies that protect against I/R injury by inhibiting ROS have been explored [28, 29]. Consistently, our previous reports revealed that the attenuation of oxidative stress using N-acetylcysteine or sodium hydrosulfide (NaSH) significantly decreased the apoptosis of neuronal cells and restored neurological and motor functions during SCIR injury [1, 9, 10]. Therefore, pharmacological therapies targeting oxidative stress may be critical for limiting the damage caused by SCIR injury.

MLN4924 is a specific inhibitor of NAE. It was initially discovered via high-throughput screening as a first-in-class anticancer agent [12, 14]. MLN4924 inhibits NAE activity by binding to NAE at the active site to form a covalent Nedd8-MLN4924 adduct and thus inactivates the neddylation pathway [30]. Beyond the antiproliferative and proapoptotic properties [12, 13], the anti-inflammatory [20, 31], cytoprotective, and antioxidant effects of MLN4924 were also demonstrated recently [32]. In addition, it was demonstrated that MLN4924 remarkably attenuated H_2_O_2_-induced neuronal damage [14]. Since oxidative stress is a leading cause of SCIR injury, we evaluated whether MLN4924 could ameliorate neuronal damage during SCIR injury. Our results indicated that treatment with MLN4924 significantly attenuated oxidative stress and neuronal cell death *in vivo* and *in vitro*. Furthermore, restored neurological and motor functions were also observed during SCIR injury after MLN4924 administration. Thus, our results demonstrated that MLN4924 could effectively protect against neurodegeneration in SCIR injury.

Sirt1 is an NAD^+^-dependent protein deacetylase and the human homolog of yeast Sir2 that has been demonstrated to affect numerous processes including, but not limited to, cell survival, differentiation, senescence, and metabolism via anti-inflammation or antioxidation [33]. Previous studies have revealed that Sirt1 mediates a wide range of cellular responses via its deacetylation activity targeting numerous transcription factors such as nuclear factor-*κ*B, forkhead box O3, p53, peroxisome proliferator-activated receptor gamma coactivator-1*α*, and hypoxia-inducible factor-1*α* [34] and directly or indirectly regulates several key nuclear receptors, including the constitutive activated receptor [35], farnesoid X receptor [36], peroxisome proliferator-activated receptor [37], and liver X receptor [38], along with numerous coregulators [39, 40]. The protective effect of Sirt1 against I/R injury has been reported in various organs [22, 24]. He et al. reported that Sirt1 protected the kidney medulla against oxidative stress injury via the induction of the cytochrome C oxidase subunit II [41]. Cardiac-specific overexpression of Sirt1 stimulates the expression of prosurvival molecules that decrease the expression of proapoptotic molecules via the deacetylation of FoxO1. This reduces oxidative damage and apoptosis as well as the extent of myocardial infarction after myocardial I/R injury [23]. Thus, the modulation of Sirt1 signaling might be a potential therapeutic strategy in the treatment of SCIR injury. In the present study, we found that SCIR or exposure to H_2_O_2_ significantly reduced Sirt1 expression. However, MLN4924 treatment rescued the process; therefore, we speculated that MLN4924 protects against neurodegeneration during SCIR and this might occur via Sirt1. Our results indicated that Sirt1 inhibition via the selective inhibitor EX527 significantly increased acetylated FoxO accumulation. Accompanied by increased oxidative stress, the protective effects of MLN4924 on neuronal apoptosis and neurological and motor functions were significantly blocked by EX527. Therefore, these data demonstrated that Sirt1 is a target molecule of MLN4924 during protection against SCIR injury.

In the present study, we found that sirt1 neddylation was notably detected after NEDD8 was overexpressed and was reduced in the presence of MLN4924. Although neddylation modification has an important role in regulating ubiquitin-mediated protein degradation [42], we did not observe an apparent change in the endogenous Sirt1 content. Therefore, we speculated that sirt1 neddylation might not play a major role in MLN4924-normalized Sirt1 expression. On the contrary, we found that the mRNA expression of Sirt1 was reduced in the presence of H_2_O_2_ incubation as well as in the spinal cord after SCIR injury, whereas the expression was abundantly restored after MLN4924 administration. This suggests that MLN4924 normalizes Sirt1 expression in part by regulating Sirt1 mRNA expression.

Although we present important findings in this study, several limitations need to be addressed. First, although the SCIR model used in this study has been widely used due to the segmental blood supply of the spine, the model has its own shortcomings, such as the occurrence of lower limb ischemia. Different modified models should be considered in future studies. Second, although no apparent side effects of MLN4924 were identified in this study regarding the concentrations used *in vivo* and *in vitro*, it must be noted that MLN4924 has both positive and negative effects. Several studies have revealed the antisurvival property of MLN4924 in both cancerous cells and normal cells [30]. Therefore, further studies investigating higher doses of MLN4924 and longer observation periods are needed to strengthen and extend our present work.

## 5. Conclusions

In conclusion, our study provides evidence that MLN4924 can exert a neuroprotective effect against oxidative stress in SCIR injury by regulating Sirt1 expression and suggests the potential application of MLN4924 in the treatment of SCIR injury.

## Data Availability

The data used to support the findings of this study are available from the corresponding author upon request.

## Supplementary Materials

Supplementary Materials Supplementary Figure 1: Sirt1 neddylation detection and quantification analysis of Sirt1 mRNA expression.

## References

[1] Xie L., Yu S., Yang K., Li C., Liang Y. (2017). Hydrogen sulfide inhibits autophagic neuronal cell death by reducing oxidative stress in spinal cord ischemia reperfusion injury. *Oxidative Medicine and Cellular Longevity*.

[2] Coselli J. S., LeMaire S. A., Miller C. C. (2000). Mortality and paraplegia after thoracoabdominal aortic aneurysm repair: a risk factor analysis. *The Annals of Thoracic Surgery*.

[3] Fang B., Li X. Q., Bao N. R. (2016). Role of autophagy in the bimodal stage after spinal cord ischemia reperfusion injury in rats. *Neuroscience*.

[4] Bischoff M. S., Di Luozzo G., Griepp E. B., Griepp R. B. (2011). Spinal cord preservation in thoracoabdominal aneurysm repair. *Perspectives in Vascular Surgery and Endovascular Therapy*.

[5] Jiang H., Xiao J., Kang B., Zhu X., Xin N., Wang Z. (2016). PI3K/SGK1/GSK3*β* signaling pathway is involved in inhibition of autophagy in neonatal rat cardiomyocytes exposed to hypoxia/reoxygenation by hydrogen sulfide. *Experimental Cell Research*.

[6] Moreira M. A., Irigoyen M. C., Saad K. R. (2016). N-acetylcysteine reduces the renal oxidative stress and apoptosis induced by hemorrhagic shock. *The Journal of Surgical Research*.

[7] Radak D., Resanovic I., Isenovic E. R. (2014). Link between oxidative stress and acute brain ischemia. *Angiology*.

[8] Hierholzer C., Billiar T. R. (2001). Molecular mechanisms in the early phase of hemorrhagic shock. *Langenbeck's Archives of Surgery*.

[9] Xie L., Wang Z., Li C., Yang K., Liang Y. (2017). Protective effect of nicotinamide adenine dinucleotide (NAD(+)) against spinal cord ischemia-reperfusion injury via reducing oxidative stress-induced neuronal apoptosis. *Journal of Clinical Neuroscience*.

[10] Xie L., Yu S., Wang Z. (2017). Nicotinamide adenine dinucleotide protects against spinal cord ischemia reperfusion injury-induced apoptosis by blocking autophagy. *Oxidative Medicine and Cellular Longevity*.

[11] Zhao Y., Xiong X., Jia L., Sun Y. (2012). Targeting Cullin-RING ligases by MLN4924 induces autophagy via modulating the HIF1-REDD1-TSC1-mTORC1-DEPTOR axis. *Cell Death & Disease*.

[12] Soucy T. A., Smith P. G., Milhollen M. A. (2009). An inhibitor of NEDD8-activating enzyme as a new approach to treat cancer. *Nature*.

[13] Milhollen M. A., Traore T., Adams-Duffy J. (2010). MLN4924, a NEDD8-activating enzyme inhibitor, is active in diffuse large B-cell lymphoma models: rationale for treatment of NF-{kappa}B-dependent lymphoma. *Blood*.

[14] Andérica-Romero A. C., Hernández-Damián J., Vázquez-Cervantes G. I., Torres I., González-Herrera I. G., Pedraza-Chaverri J. (2016). The MLN4924 inhibitor exerts a neuroprotective effect against oxidative stress injury via Nrf2 protein accumulation. *Redox Biology*.

[15] Yan M., Yang M., Shao W. (2014). High-dose ascorbic acid administration improves functional recovery in rats with spinal cord contusion injury. *Spinal Cord*.

[16] Shiroyama Y., Nagamitsu T., Yamashita K., Yamashita T., Abiko S., Ito H. (1991). An examination of change in blood flow in the brainstem of rats under various grades of ischemia. *No Shinkei Geka*.

[17] Basso D. M., Beattie M. S., Bresnahan J. C. (1995). A sensitive and reliable locomotor rating scale for open field testing in rats. *Journal of Neurotrauma*.

[18] Chen Z., Zhang Z., Zhang D., Li H., Sun Z. (2016). Hydrogen sulfide protects against TNF-*α* induced neuronal cell apoptosis through miR-485-5p/TRADD signaling. *Biochemical and Biophysical Research Communications*.

[19] Li L., Jiang H. K., Li Y. P., Guo Y. P. (2015). Hydrogen sulfide protects spinal cord and induces autophagy via miR-30c in a rat model of spinal cord ischemia-reperfusion injury. *Journal of Biomedical Science*.

[20] Liu K., Chen K., Zhang Q. (2019). TRAF6 neddylation drives inflammatory arthritis by increasing NF-*κ*B activation. *Laboratory Investigation*.

[21] Chai W., Wang Y., Lin J. Y. (2012). Exogenous hydrogen sulfide protects against traumatic hemorrhagic shock via attenuation of oxidative stress. *The Journal of Surgical Research*.

[22] Nakamura K., Zhang M., Kageyama S. (2017). Macrophage heme oxygenase-1-SIRT1-p53 axis regulates sterile inflammation in liver ischemia-reperfusion injury. *Journal of Hepatology*.

[23] Hsu C. P., Zhai P., Yamamoto T. (2010). Silent information regulator 1 protects the heart from ischemia/reperfusion. *Circulation*.

[24] Lin Y., Sheng M., Ding Y. (2018). Berberine protects renal tubular cells against hypoxia/reoxygenation injury via the Sirt1/p53 pathway. *Journal of Natural Medicines*.

[25] Lombardi V., Valko L., Štolc S. (1998). Free radicals in rabbit spinal cord ischemia: electron spin resonance spectroscopy and correlation with SOD activity. *Cellular and Molecular Neurobiology*.

[26] Temiz C., Solmaz I., Tehli O. (2013). The effects of splenectomy on lipid peroxidation and neuronal loss in experimental spinal cord ischemia/reperfusion injury. *Turkish Neurosurgery*.

[27] Usul H., Cakir E., Cobanoglu U. (2004). The effects of tyrphostine Ag 556 on experimental spinal cord ischemia reperfusion injury. *Surgical Neurology*.

[28] Kim J. K., Pedram A., Razandi M., Levin E. R. (2006). Estrogen prevents cardiomyocyte apoptosis through inhibition of reactive oxygen species and differential regulation of p38 kinase isoforms. *The Journal of Biological Chemistry*.

[29] Shiva S., Sack M. N., Greer J. J. (2007). Nitrite augments tolerance to ischemia/reperfusion injury via the modulation of mitochondrial electron transfer. *The Journal of Experimental Medicine*.

[30] Li L., Liu B., Dong T. (2013). Neddylation pathway regulates the proliferation and survival of macrophages. *Biochemical and Biophysical Research Communications*.

[31] Chang F. M., Reyna S. M., Granados J. C. (2012). Inhibition of neddylation represses lipopolysaccharide-induced proinflammatory cytokine production in macrophage cells. *The Journal of Biological Chemistry*.

[32] Scudder S. L., Patrick G. N. (2015). Synaptic structure and function are altered by the neddylation inhibitor MLN4924. *Molecular and Cellular Neurosciences*.

[33] Haigis M. C., Guarente L. P. (2006). Mammalian sirtuins--emerging roles in physiology, aging, and calorie restriction. *Genes & Development*.

[34] Ryu D. R., Yu M. R., Kong K. H. (2019). Sirt1-hypoxia-inducible factor-1*α* interaction is a key mediator of tubulointerstitial damage in the aged kidney. *Aging Cell*.

[35] Corton J. C., Brown-Borg H. M. (2005). Peroxisome proliferator-activated receptor *γ* coactivator 1 in caloric restriction and other models of longevity. *The Journals of Gerontology Series A: Biological Sciences and Medical Sciences*.

[36] Kemper J. K., Choi S. E., Kim D. H. (2013). Chapter sixteen - sirtuin 1 deacetylase: a key regulator of hepatic lipid metabolism. *Obesity*.

[37] Purushotham A., Schug T. T., Xu Q., Surapureddi S., Guo X., Li X. (2009). Hepatocyte-specific deletion of SIRT1 alters fatty acid metabolism and results in hepatic steatosis and inflammation. *Cell Metabolism*.

[38] Li X., Zhang S., Blander G., Tse J. G., Krieger M., Guarente L. (2007). SIRT1 deacetylates and positively regulates the nuclear receptor LXR. *Molecular Cell*.

[39] Schug T. T., Li X. (2011). Sirtuin 1 in lipid metabolism and obesity. *Annals of Medicine*.

[40] Zhao Q., Liu F., Cheng Y. (2019). Celastrol protects from cholestatic liver injury through modulation of SIRT1-FXR signaling. *Molecular & Cellular Proteomics*.

[41] He W., Wang Y., Zhang M. Z. (2010). Sirt1 activation protects the mouse renal medulla from oxidative injury. *The Journal of Clinical Investigation*.

[42] Gao F., Cheng J., Shi T., Yeh E. T. H. (2006). Neddylation of a breast cancer-associated protein recruits a class III histone deacetylase that represses NF*κ*B-dependent transcription. *Nature Cell Biology*.

