# Supplementary material for: MLN4924 Exerts a Neuroprotective Effect against Oxidative Stress via Sirt1 in Spinal Cord Ischemia-Reperfusion Injury
Source: Oxid Med Cell Longev. 2019 Apr 17;2019:7283639. doi: 10.1155/2019/7283639 (PMC6501157; doi:10.1155/2019/7283639)
Supplement: Supplementary Materials — Supplementary Figure 1: Sirt1 neddylation detection and quantification analysis of Sirt1 mRNA expression. [file 7283639.f1.pdf]

Supplementary figure 1. Sirt1 neddylation detection and quantification analysis of Sirt1 mRNA expression

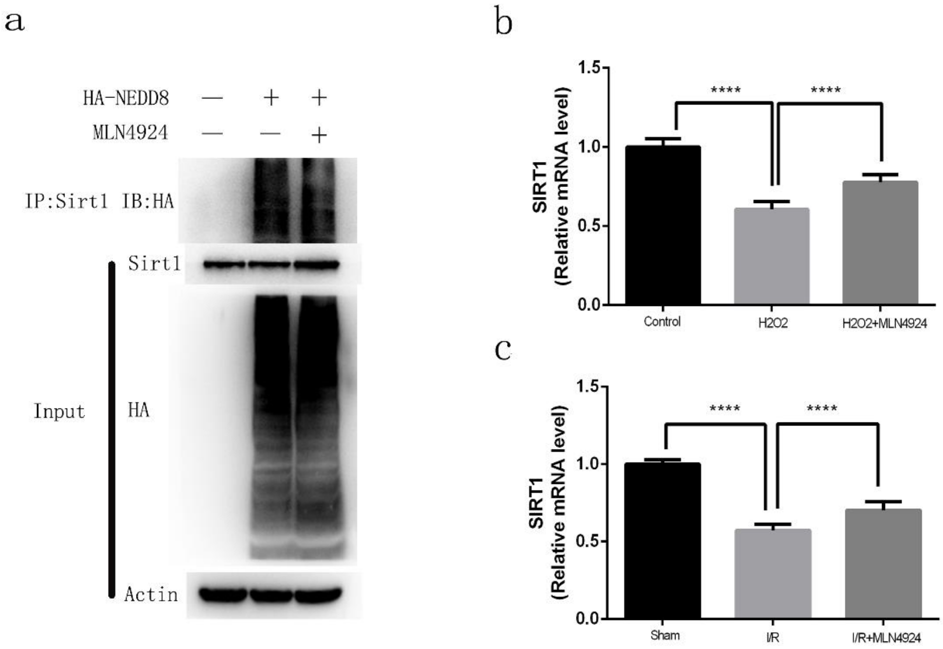

Supplementary figure 1. Sirt1 neddylation detection and quantification analysis of Sirt1 mRNA expression. (a) Sirt1 neddylation in SH-SY5Y cells after NEDD8 overexpression treated with or without MLN4924. (b) Quantification analysis of Sirt1 mRNA expression in SH-SY5Y cells treated with or without H_2O_2/MLN4924. (c) Quantification analysis of Sirt1 mRNA expression in spinal cord of rats treated with or without MLN4924 after SCIR injury. N=6. ****$P<0.0001$. *P*-values were analyzed by one-way ANOVA.
